# Comparison of chest computed tomography features between pulmonary tuberculosis patients with culture-positive and culture-negative sputum for non-mycobacteria: A retrospective observational study

**DOI:** 10.1097/MD.0000000000026897

**Published:** 2021-08-06

**Authors:** Takamasa Kan, Kosaku Komiya, Mari Yamasue, Mariko Itai, Ai Tanaka, Yukiko Takeno, Shuichi Takikawa, Kazufumi Hiramatsu, Jun-ichi Kadota

**Affiliations:** a Department of Respiratory Medicine and Infectious Diseases, Oita University Faculty of Medicine, 1-1 Idaigaoka, Hasama-machi, Yufu, Oita, Japan; b Department of Internal Medicine, National Hospital Organization Nishi-Beppu Hospital, 4548 Tsurumi, Beppu, Oita, Japan.

**Keywords:** chest CT, pulmonary tuberculosis, sputum

## Abstract

Although complication with non-mycobacterial pneumonia among patients with pulmonary tuberculosis (TB) may lead to poor prognosis, discrimination between TB complicated with and without non-mycobacterial pneumonia using radiological imaging has not been fully elucidated. We aimed to clarify the differences in chest computed tomography (CT) features between pulmonary TB patients with culture-positive and culture-negative sputum for non-mycobacteria.

We retrospectively included consecutive patients admitted to our hospital from January 2013 to December 2015 for bacteriologically-confirmed pulmonary TB, who were tested by sputum culture for non-mycobacteria, and who underwent chest CT within 2 weeks before or after admission. Chest CT features were compared between pulmonary TB patients who had positive non-mycobacterial cultures and in those who had not.

Of 202 patients with pulmonary TB, 186 (92%) were tested by sputum culture for non-mycobacteria and underwent chest CT. Among these, non-mycobacteria were isolated in 118 patients (63%), while 68 patients (37%) had negative cultures. Patients with a positive culture for non-mycobacteria were significantly older and had lower levels of physical activity and albumin, higher levels of C-reactive protein, and a greater number of respiratory failures. By CT, emphysematous lesions, ground-glass opacities, airspace consolidation, air-bronchogram, interlobular septal thickening, bronchiectasis, pleural effusion, pleural thickening, and lymph node enlargement were more frequently in patients with a positive culture for non-mycobacteria. These chest CT features could be helpful for detecting complication with non-mycobacterial pneumonia in patients with pulmonary TB.

## 1 Introduction

While the prevalence and incidence of tuberculosis (TB) have been declining worldwide, the incidence of TB reflecting endogenous reactivation after initial TB infection in the past remains high in high- or middle-income countries, in which the elderly population is increasing.^[1,2]^

Advanced age is known to be a risk factor not only for the development of TB, but also for community-acquired non-mycobacterial pneumonia.^[3,4]^ Pulmonary tuberculosis can be complicated with non-mycobacterial pneumonia (30%–62%),^[5,6]^ and this condition may lead to poor prognosis. Nevertheless, routine non-mycobacterial culture is not officially recommended for TB patients.^[7]^ Although complication with non-mycobacterial pneumonia in patients with pulmonary TB can be considered based on the results of laboratory or radiological tests, the features that can be ascertained by chest computed tomography (CT) to distinguish pulmonary TB patients with and without complication by non-mycobacterial pulmonary infection remain unclear. In our institute, we routinely culture sputum samples for non-mycobacteria and undertake chest CT on admission for patients with pulmonary TB. Hence, this study aimed to clarify the differences in chest CT features between pulmonary TB patients with and without positive sputum cultures for non-mycobacteria.

## 2 Materials and methods

### 2.1 Patients and study design

This was a retrospective observational study performed at the National Hospital Organization Nishi-Beppu Hospital, the only hospital with the capacity to accept patients with smear-positive pulmonary TB in the Oita Prefecture, Japan. We included consecutive patients admitted to the hospital between January 2013 and December 2015 for bacteriologically-confirmed pulmonary TB, who concurrently had sputum samples cultured for non-mycobacteria, and who underwent chest CT within 2 weeks before or after admission.

TB patients were classified into 2 groups; the culture-positive and the culture-negative for non-mycobacteria groups. Patients with sputum samples with commensal flora were regarded as culture-negative for non-mycobacteria. The clinical characteristics and chest CT features between these 2 groups were compared. The study protocol was approved by the institutional ethics committee (approval number, 1–14; approval date, March 27, 2020). Informed consent was waived by the committee because of the fact that the study was a retrospective study, and information on this study was posted at the hospital, with a method to opt out. Some of the patients included in this study had already participated in previous studies,^[2,6,8,9]^ but the aims of the previous studies and this study did not overlap.

### 2.2 Data collection

Patient data, including data on gender, age, body mass index, daily physical activity levels, underlying diseases, laboratory data, and presence of respiratory failure, were obtained from clinical records. The collection of this patient information and examination of pulmonary performance are routinely recommended when a patient diagnosed with pulmonary TB is admitted to our hospital. We evaluated daily physical activity on admission using a scale of performance status.^[10]^ The definition of respiratory failure was SpO_2_ < 90% without oxygen inhalation on admission.

### 2.3 Evaluation of chest computed tomography findings

A 16-detector rows CT scanner (Activion, Toshiba Medical Systems, Tokyo, Japan) was used at the study hospital. Scans were obtained using 1.0-mm-thick sections of contiguous images from the apex to the lung base. Images were photographed at a window setting of –600 HU (level) and 1600 HU (width). If the patient underwent CT before referral to our hospital, we evaluated the CT features from the images taken at the referring institutes.

Two respiratory medicine specialists with 9 and 13 years of experience (KT and MY), who were blinded to laboratory data, clinical features, and patient diagnosis, independently evaluated chest CT features, which included emphysematous lesions, ground-glass opacities, airspace consolidation, nodules, granulomas, air-bronchogram, cavitary lesions, interlobular septal thickening, and bronchiectasis. Investigators ascertained the distributions of these features in each 3 areas in both lungs regarding the lingular segment as the middle area in the left lung. The presence of lung involvement was defined as occupation of more than 50% of each lung area.

Furthermore, chest CT images reconstructed using the mediastinal setting were used to evaluate the presence of other CT features such as pleural effusion, pleural thickening, mediastinum, and hilar lymphadenopathy (with a short-axis diameter of larger than 1 cm) and calcification of those lymph nodes. Any disagreement between the presence of these findings in each case was resolved by a review conducted by the same 2 physicians in order to reach a consensus.

### 2.4 Statistical analyzes

Statistical analyzes were performed using the IBM SPSS statistics version 24 software package (IBM Japan, Tokyo, Japan). Logistic regression analysis was used for patients in whom non-mycobacteria were isolated. Statistical significance was defined as a probability (*P*) value <.05 for all analyses. Interobserver agreement was assessed by kappa value analysis. For two-tailed analyzes, 95% confidence intervals were calculated, and a *P* value <.05 was considered to indicate statistical significance for all analyzes.

## 3 Results

### 3.1 Baseline characteristics between patients with a positive culture and a negative culture for non-mycobacteria

**Figure 1:**
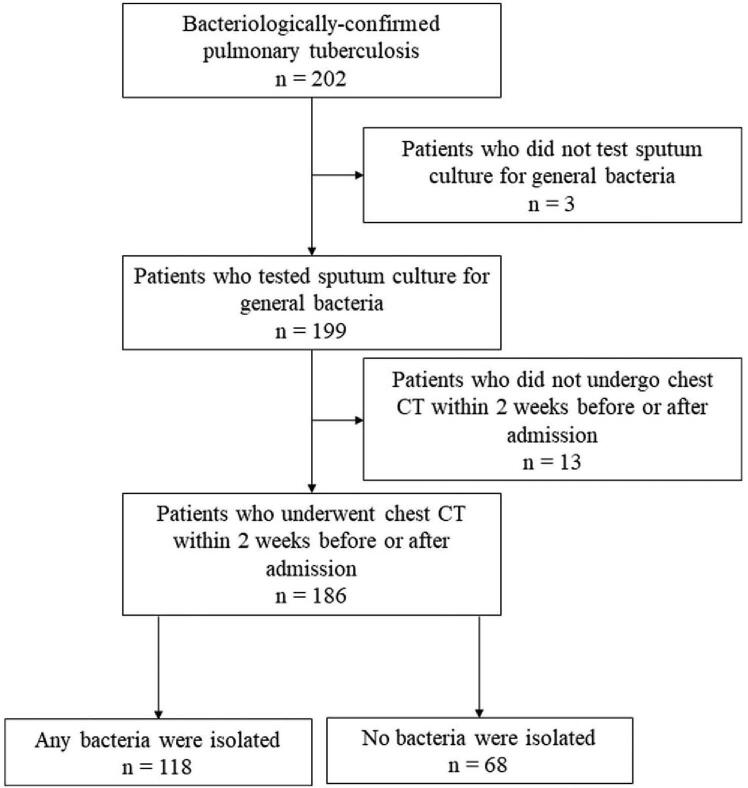
A flow chart of the participants evaluated over the course of the study and the number of patients in each group.

Of 202 patients with bacteriologically-confirmed pulmonary TB, 199 (99%) had sputum samples cultured for non-mycobacteria. Among these patients, 186 (93%) underwent chest CT within 2 weeks before or after admission (Fig. 1). Hence, this study eventually included 118 patients with a positive culture for non-mycobacteria (63%) and 68 (37%) patients with a negative culture for non-mycobacteria. Almost half (51%) of the patients were women, and the median age was 82 (interquartile range, 73–88). While *Mycobacterium tuberculosis* with resistance to more than one first-line anti-TB drug (i.e., isoniazid, rifampin, ethambutol, and pyrazinamide) was detected in 14 patients (8%), no resistance to both isoniazid and rifampin was observed. Thirty nine patients (21%) died during hospitalization. Patients in the culture-positive group were significantly older and had a greater number of respiratory failures, poorer performance status, lower levels of hemoglobin and albumin, and higher levels of aspartate aminotransferase, alanine aminotransferase, and C-reactive protein (CRP) than the patients in the culture-positive group (Table 1).

**Table 1:** Baseline characteristics of pulmonary tuberculosis (TB) patients with a positive culture and a negative culture for non-mycobacteria.

	Culture-positive for non-mycobacteria (n = 118)	Culture-negative for non-mycobacteria (n = 68)	OR	95% CI	*P* value
Female gender	57 (48)	37 (54)	0.783	0.430–1.424	.423
Age (years old)	83 (77–89)	79 (62–84)	1.045	1.023–1.068	<.001
BMI (kg/m^2^)	18.6 (16.4–20.5)	19.8 (17.9–21.4)	0.877	0.793–0.971	.011
Performance status	3 (2–4)	2 (1–3)	1.890	1.443–2.475	<.001
Diabetes mellitus	20 (17)	14 (21)	0.787	0.368–1.682	.537
Cerebrovascular disease	21 (18)	9 (13)	1.419	0.609–3.305	.417
Heart failure	21 (18)	8 (12)	1.621	0.683–3.939	.268
COPD	7 (6)	4 (6)	1.009	0.284–3.580	.989
Chronic kidney disease	13 (11)	5 (7)	1.560	0.531–4.583	.419
Hepatic disease	8 (7)	6 (9)	0.758	0.252–2.286	.623
Respiratory failure on admission	43 (36)	9 (13)	3.313	1.487–7.378	.003
Respiratory failure during hospitalization	63 (53)	16 (24)	3.723	1.911–7.253	<.001
WBC (×10^3^/μL)	6.6 (5.2–9.1)	6.9 (5.1–8.5)	1.005	0.913–1.107	.919
Hemoglobin (g/dL)	11.0 (9.9–12.2)	12.5 (10.9–13.6)	0.797	0.687–0.926	.003
Albumin (g/dL)	2.6 (2.1–3.2)	3.2 (2.6–3.6)	0.410	0.257–0.652	<.001
CRP (mg/dL)	5.1 (1.3–10.2)	1.6 (0.5–3.8)	1.181	1.089–1.282	<.001
AST (IU/L)	28.0 (22.0–41.3)	21.0 (18.0–29.3)	1.020	1.003–1.037	.021
ALT (IU/L)	18.5 (10.0–30.0)	15.0 (10.0–23.0)	1.015	1.000–1.031	.043
BUN (mg/dL)	16.7 (13.6–25.1)	15.0 (10.9–19.5)	1.021	0.996–1.047	.105
Creatinine (mg/dL)	0.70 (0.50–0.91)	0.75 (0.57–0.87)	0.841	0.392–1.805	.656
Smear grade	1 (1–2)	1 (1–2)	1.622	0.933–2.822	.087
Time to negative conversion (day)	49 (33–71)	45 (29–64)	1.006	0.993–1.018	.368
In-hospital death	32 (27)	7 (10)	3.243	1.343–7.827	.009

### 3.2 Sputum culture results and additional antibiotics for patients with a positive culture for non-mycobacteria

*Staphylococcus aureus*, including methicillin-resistant *S. aureus*, was most commonly isolated from the sputum samples, followed in frequency by *Klebsiella pneumoniae*, *Staphylococcus haemolyticus*, and *Enterobacter cloacae* (Table 2). Additional antibiotics were administered for 65 patients (55%) in the culture-positive group. Levofloxacin was most frequently used (25 patients), and antibiotics covering methicillin-resistant *Staphylococcus aureus* and *Pseudomonas aeruginosa* were used in 2 (2%) and 43 (66%) of the 65 cases, respectively. In-hospital mortality in the culture-positive group was significantly higher than that in the culture-negative group (Table 1), and culture-positive patients who were treated with additional antibiotics had lower in-hospital mortality than those who were not (42% vs 10%, *P* < .001). There were no sepsis cases caused by non-mycobacteria.

**Table 2:** Non-mycobacteria isolated in the sputum of pulmonary tuberculosis (TB) patients.

Bacterial species	Number
Staphylococcus aureus	46 (39)
Methicillin-resistant Staphylococcus aureus	33 (28)
Methicillin-susceptible Staphylococcus aureus	13 (11)
Klebsiella pneumoniae	18 (15)
Staphylococcus haemolyticus	16 (14)
Enterobacter cloacae	12 (10)
Pseudomonas aeruginosa	11 (9)
Staphylococcus epidermidis	8 (7)
Stenotrophomonas maltophilia	7 (6)

### 3.3 Comparison of chest computed tomography features between patients with a positive culture and a negative culture for non-mycobacteria

The kappa values of the CT findings were as follows: 0.89 for emphysematous lesion, 0.75 for ground-glass attenuation, 0.89 for airspace consolidation, 0.69 for nodule, 0.71 for granuloma, 0.93 for air-bronchogram, 0.87 for cavitary lesion, 0.84 for interlobular septal thickening, 0.79 for bronchiectasis, 0.90 for pleural effusion, 0.92 for pleural thickening, 0.91 for lymphadenopathy, and 0.96 for lymph node calcification.

As shown in Table 3, emphysematous lesions, ground-glass opacities, airspace consolidation, air-bronchogram, interlobular septal thickening, bronchiectasis, pleural effusion, pleural thickening, lymphadenopathy and calcification of those lymph nodes were more frequently observed in patients with a positive culture for non-mycobacteria than in those without. The number of lobes involved in the culture-positive group was significantly larger than that in the no bacteria-isolated group. These infiltrates were more commonly distributed in both lungs, right lung, and middle lobes in the culture-positive group.

**Table 3:** Chest CT features of pulmonary tuberculosis (TB) in patients with a positive culture and a negative culture for non-mycobacteria.

	Culture-positive for non-mycobacteria (n = 118)	Culture-negative for non-mycobacteria (n = 68)	OR	95% CI	*P* value
Emphysematous lesions	28 (24)	6 (9)	3.215	1.257–8.223	.015
Ground glass opacities	50 (42)	17 (25)	2.206	1.141–4.265	.019
Airspace consolidation	90 (76)	40 (59)	2.250	1.183–4.279	.013
Nodule	72 (61)	44 (65)	0.854	0.459–1.587	.617
Granuloma	103 (87)	62 (91)	0.665	0.245–1.802	.422
Air-bronchogram	81 (69)	34 (50)	2.189	1.185–4.046	.012
Cavity	59 (50)	32 (47)	1.125	0.619–2.045	.699
Interlobular septal thickening	36 (31)	10 (15)	2.546	1.171–5.539	.018
Bronchiectasis	69 (59)	26 (38)	2.275	1.235–4.191	.008
Pleural effusion	49 (42)	15 (22)	2.509	1.271–4954	.008
Pleural thickening	57 (48)	18 (27)	2.596	1.357–4.965	.004
Enlargement of mediastinum and/or hilar lymph node	67 (57)	25 (37)	2.260	1.224–4.171	.009
Lymph node calcification	42 (36)	14 (21)	2.132	1.060–4.285	.034
Number of lobe involvements	3 (1–4)	1 (1–3)	1.291	1.075–1.550	.006
Distributions					
Bilateral lungs	64 (54)	22 (32)	2.478	1.328–4.625	.004
Right lung	106 (90)	51 (75)	2.944	1.309–6.625	.009
Left lung	76 (64)	39 (57)	1.346	0.731–2.478	.341
Upper lobes	96 (81)	52 (77)	1.343	0.649–2.778	.427
Middle lobes	65 (55)	26 (38)	1.981	1.078–3.642	.028
Lower lobes	75 (64)	34 (50)	1.744	0.952–3.195	.072

## 4 Discussion

This study demonstrated that emphysematous lesion, ground-glass opacity, airspace consolidation, air-bronchogram, interlobular septal thickening, bronchiectasis, pleural effusion, pleural thickening, and lymph node enlargement were more frequently observed, and that these more commonly distribute to the bilateral lungs, right lung, and middle lobes in pulmonary TB patients with a positive culture for non-mycobacteria than in those without.

Patients with a positive culture for non-mycobacteria had a lower physical activity level and poorer nutritional status than those without. These patient background data are known to be risk factors for aspiration pneumonia,^[11]^ so patients with a positive culture for non-mycobacteria might have developed pneumonia through aspiration of oral secretions. CRP levels were significantly elevated in the culture-positive group compared with those in the culture-negative group. This observations appears consistent with the fact that non-mycobacterial infection would usually increase the CRP level, whereas TB infection would not.^[12,13]^ CRP levels might be a clue to distinguishing complication by non-mycobacterial infection among patients with pulmonary TB.

Typical bacterial pathogens that cause community-acquired non-mycobacterial pneumonia mainly include *Streptococcus pneumoniae*, *Haemophilus influenzae*, and *Moraxella catarrhalis*.^[14]^ However, in this study, *S. aureus* was most commonly isolated, followed by *K. pneumoniae*. These pathogens are known to colonize the oral cavity and can be aspirated to peripheral airways; thus, these results support that patients with a positive culture for non-mycobacteria might have aspirated oral secretions. Furthermore, additional antibiotics were administered for 65 patients (55%) in this group. Whether these patients should be treated with additional antibiotics needs to be determined. Isolated bacteria are not always true causative pathogens; thus, some physicians hesitate to add antibiotics to anti-TB drugs. It is also noted that rifampicin has antibacterial activity against not only *M. tuberculosis* but also other common bacteria,^[15]^ which may discourage physicians from administering additional antibiotics. Indeed, additional antibiotics seem not to improve the prognosis of pulmonary TB patients whose sputum culture was positive for non-mycobacteria.^[6]^ In our study, culture-positive patients who were treated with additional antibiotics had lower in-hospital mortality than those who were not. Physicians have presumably administered additional antibiotics for severe patients.

Ground-glass opacity, airspace consolidation, and air-bronchogram were more frequently seen in patients with a positive culture for non-mycobacteria than in those without. These findings are common features in community-acquired non-mycobacterial pneumonia, but the incidence in pulmonary TB patients warrants discussion. While primary pulmonary TB may cause exudative inflammation, secondary pulmonary TB tends to be characterized by an endobronchial spread of infection, exhibiting the tree-in-bud sign.^[16,17]^ The present study included a large number of elderly patients, and most of these could have developed secondary TB. This patient background may explain why patients with a negative culture for non-mycobacteria were less likely to have ground-glass opacity, airspace consolidation, and air-bronchogram that reflected exudative inflammation.

Emphysematous lesions and bronchiectasis were more commonly observed in patients with a positive culture for non-mycobacteria, probably because these underlying conditions were significant risk factors for bacterial colonization and might be associated with the development of non-mycobacterial pneumonia.^[18]^ However, it is challenging to determine whether these non-mycobacteria were cultured as infection or colonization. Interlobular septal thickening and pleural effusion could be a consequence of congestive pulmonary edema, which is thought to be induced by hyperinflammation.^[19,20]^ Complication with non-mycobacterial infection might accelerate the pulmonary edema. Similarly, lymph node enlargement, which was more frequently observed in patients with a positive culture for non-mycobacteria, could be caused by hyperinflammation due to non-mycobacterial bacterial infection.^[21]^ The results that no differences were observed in nodule and granuloma between 2 groups may be reasonable because these features would less likely reflect ongoing infections.

Infiltrates were more commonly observed in both lungs, the right lung, and middle lobes in patients with a positive culture for non-mycobacteria. We have previously shown that patients with poor performance status may show atypical distribution of lung involvement (i.e., not limited to the upper lobes).^[2]^ Considering the observation that patients with a positive culture for non-mycobacteria had a lower physical activity level than patients with a negative culture for non-mycobacteria, it would be reasonable that culture-positive patients are prone to extensive infiltrate distributions. The usual site of aspiration pneumonia is the lower lobe of the right lung. The fact that a right-lung distribution was more frequently observed in patients with a positive culture for non-mycobacteria is consistent with pneumonia caused by aspiration.

The strength of this study is that it is the first to compare chest CT features between patients with a positive culture for non-mycobacteria and those without. The fact that in our hospital, most elderly patients with pulmonary TB requiring hospitalization routinely submit sputum samples for non-mycobacterial culture and undergo chest CT on admission is also an advantage of this study, despite its retrospective design. However, some limitations of this study should also be considered. First, whether isolated non-mycobacteria reflected colonization or infection could not be determined, as no clear definition to discriminate these conditions exists. In addition, the culture results might be associated with laboratory capacity. Technicians in our institute do not routinely evaluate the quality of the sputum. The second limitation is a matter of potentially limited generalizability: Since our study population included a large number of elderly patients, our observations and conclusions might not be applicable to younger populations of TB patients. Third, interobserver agreements for a few chest CT features were insufficient.

In conclusion, this study revealed significant differences in chest CT features between pulmonary TB patients with and without culture-positive sputum samples for non-mycobacteria. These findings might be helpful to distinguish pulmonary TB patients complicated with non-mycobacterial pneumonia from those without.

## Acknowledgments

The authors thank Dr. Hiroshi Kawano, Dr. Masahide Hara, and Dr. Kazuya Goto (National Hospital Organization Nishi-beppu Hospital, Oita) for their advice and support.

## Author contributions

**Conceptualization:** Takamasa Kan, Kosaku Komiya, Mari Yamasue, Mariko Itai, Yukiko Takeno, Shuichi Takikawa, Kazufumi Hiramatsu, Jun-ichi Kadota.

**Data curation:** Takamasa Kan, Mari Yamasue, Mariko Itai, Ai Tanaka, Yukiko Takeno, Shuichi Takikawa.

**Formal analysis:** Takamasa Kan, Kosaku Komiya, Mari Yamasue, Ai Tanaka, Yukiko Takeno, Shuichi Takikawa.

**Investigation:** Takamasa Kan, Kosaku Komiya, Shuichi Takikawa.

**Methodology:** Takamasa Kan, Kosaku Komiya, Mari Yamasue, Mariko Itai, Ai Tanaka, Shuichi Takikawa.

**Project administration:** Takamasa Kan, Kosaku Komiya, Mari Yamasue, Shuichi Takikawa, Jun-ichi Kadota.

**Resources:** Ai Tanaka, Shuichi Takikawa.

**Supervision:** Kosaku Komiya, Shuichi Takikawa, Kazufumi Hiramatsu, Jun-ichi Kadota.

**Validation:** Mariko Itai.

**Visualization:** Ai Tanaka, Yukiko Takeno.

**Writing – original draft:** Takamasa Kan, Kosaku Komiya, Mari Yamasue, Mariko Itai, Shuichi Takikawa, Kazufumi Hiramatsu, Jun-ichi Kadota.

**Writing – review & editing:** Takamasa Kan, Kosaku Komiya, Mari Yamasue, Mariko Itai, Kazufumi Hiramatsu, Jun-ichi Kadota.
